# Practical Electricity in Medicine and Surgery

**Published:** 1890-02

**Authors:** 


PRACTICAL ELECTRICITY IN MEDICINE AND SURGERY.—By G. A. Liebig, Jr., Ph. D., Assistant in Electricity Johns Hopkins University, Etc., Etc., and George H. Rohe, M. D., Professor of Obstetrics and Hygiene, College of Physicians and Surgeons, Baltimore, Etc., Etc., profusely illustrated. Philadelphia and London. F. A. Davis, Publisher, 1890.

The object of the authors in preparing this admirable treatise, was to set forth in a concise manner, the fundamental principles which are involved in the application of electricity to medical and surgical practice, and their attempt has been in the highest degree successful. Part I, describes the various forms of electrical and magnetic apparatus used by the physician in his daily experience with electricity, the construction and use of galvanometers, the theory of the chemical actions of the storage cell, and the best methods of caring for such batteries. Reference is also made to the electric motor, telephone and phonograph. Part II, refers to the effects of electric currents on the various tissues and organs of the body in health, and then explains how these effects are modified by disease, and indicates the methods by which such modifications are utilized for purposes of diagnosis. Also the appliances most useful in electro-therapeutic work. Part III, describes the application of electricity in the treatment of disease, and the methods by which it is made available for therapeutic purposes. Particular attention is also given to the application of electricity in gynæcology, the diseases of the male genito, urinary organs, and in diseases of the skin, the whole comprising an intelligent account of the science of electricity, and a trustworthy guide to its applications in the practice of medicine and surgery, and forming a valuable contribution to medical science.

